# Supplementary material for: A UMLS-based spell checker for natural language processing in vaccine safety
Source: BMC Med Inform Decis Mak. 2007 Feb 12;7:3. doi: 10.1186/1472-6947-7-3 (PMC1805499; doi:10.1186/1472-6947-7-3)
Supplement: Additional file 13 — RAP application directory. Sets up directory for RAP (RDF application for PHP) source code [file 1472-6947-7-3-S13.gz › rap/doc/phpdoc/__filesource/fsource_utility__utilRdfUtil.php.html]

xml version="1.0" encoding="iso-8859-1"?


File Source for RdfUtil.php


# Source for file RdfUtil.php

Documentation is available at RdfUtil.php

```
1. <?php

3. // ----------------------------------------------------------------------------------
4. // Class: RDFUtil
5. // ----------------------------------------------------------------------------------


9. /**
10. * Useful utility methods.
11. * Static class.
12. *
13. * <BR><BR>History:<UL>
14. * <LI>03-29-2005                 : Function guessNamespace() and visualizeGraph() added (anton1@koestlbacher.de)</LI>
15. * <LI>12-06-2004                 : improved namespace handling in function</LI>
16. *                                  writeAsHTMLTable() added (tobias.gauss@web.de)</LI>
17. * <LI>09-10-2004                 : added support for OWL and infered statements</LI>
18. * <LI>11-18-2003                 : Function writeAsHtmlTable() htmlspecialchars & nl2br
19. *                                   for displaying literals added.</LI>
20. * <LI>04-23-2003                 : Chunk_split() removed from writeHTMLTable</LI>
21. * <LI>12-04-2002                 : Added support for "rdf:datatype" in writeHTMLTable</LI>
22. * <LI>10-03-2002                : Green coloring for RDF_SCHEMA nodes added to writeHTMLTable</LI>
23. * <LI>09-10-2002                : First version of this class.</LI>
24. *
25. * </UL>
26. * @version  V0.9.1
27. * @author Chris Bizer <chris@bizer.de>, Daniel Westphal <dawe@gmx.de>
28. * @author   Anton K�stlbacher <anton1@koestlbacher.de>
29. * @package utility
30. * @access    public
31. */
32. class RDFUtil extends Object {

34. /**
35. * Extracts the namespace prefix out of a URI.
36. *
37. * @param    String    $uri
38. * @return    string
39. * @access    public
40. */
41. function guessNamespace($uri) {
42. $l = RDFUtil::getNamespaceEnd($uri);
43. return $l > 1 ? substr($uri ,0, $l) : "";
44. }

46. /**
47. * Delivers the name out of the URI (without the namespace prefix).
48. *
49. * @param    String    $uri
50. * @return    string
51. * @access    public
52. */
53. function guessName($uri) {
54. return substr($uri,RDFUtil::getNamespaceEnd($uri));
55. }

57. /**
58. * Extracts the namespace prefix out of the URI of a Resource.
59. *
60. * @param    Object Resource    $resource
61. * @return    string
62. * @access    public
63. */
64. function getNamespace($resource) {
65. return RDFUtil::guessNamespace($resource->getURI());
66. }

68. /**
69. * Delivers the Localname (without the namespace prefix) out of the URI of a Resource.
70. *
71. * @param    Object Resource    $resource
72. * @return    string
73. * @access    public
74. */
75. function getLocalName($resource) {
76. return RDFUtil::guessName($resource->getURI());
77. }

79. /**
80. * Position of the namespace end
81. * Method looks for # : and /
82. * @param    String    $uri
83. * @access    private
84. */
85. function getNamespaceEnd($uri) {
86. $l = strlen($uri)-1;
87. do {
88. $c = substr($uri, $l, 1);
89. if($c == '#' || $c == ':' || $c == '/')
90. break;
91. $l--;
92. } while ($l >= 0);
93. $l++;
94. return $l;
95. }

97. /**
98. * Short Prefix for known Namespaces by given URI
99. * @param    String    $uri
100. * @access    public
101. */
102. function getPrefix($uri) {
103. switch (RDFUtil::guessNamespace($uri))
104. {
105. case RDF_NAMESPACE_URI:
106. $prefix = RDF_NAMESPACE_PREFIX;
107. break;

109. case RDF_SCHEMA_URI:
110. $short_p = RDF_SCHEMA_PREFIX;
111. break;

113. case OWL_URI:
114. $short_p = OWL_PREFIX;
115. break;

117. default:
118. $short_p = $statement->getLabelPredicate();
119. }
120. return $short_p;
121. }


124. /**
125. * Tests if the URI of a resource belongs to the RDF syntax/model namespace.
126. *
127. * @param    Object Resource    $resource
128. * @return    boolean
129. * @access    public
130. */
131. function isRDF($resource) {
132. return ($resource != NULL && RDFUtil::getNamespace($resource) == RDF_NAMESPACE_URI);
133. }

135. /**
136. * Escapes < > and &
137. *
138. * @param    String    $textValue
139. * @return    String
140. * @access    public
141. */
142. function escapeValue($textValue) {

144. $textValue = str_replace('<', '&lt;', $textValue);
145. $textValue = str_replace('>', '&gt;', $textValue);
146. $textValue = str_replace('&', '&amp;', $textValue);

148. return $textValue;
149. }

151. /**
152. * Converts an ordinal RDF resource to an integer.
153. * e.g. Resource(RDF:_1) => 1
154. *
155. * @param    object Resource    $resource
156. * @return    Integer
157. * @access    public
158. */
159. function getOrd($resource)  {
160. if($resource == NULL || !is_a($resource, 'Resource') || !RDFUtil::isRDF($resource))
161. return -1;
162. $name = RDFUtil::getLocalName($resource);
163. echo substr($name, 1).' '.RDFUtil::getLocalName($resource);
164. $n = substr($name, 1);
165. //noch rein : chekcen ob $n Nummer ist !!!!!!!!!!!!!!!!!!!!!!if($n)
166. return $n;
167. return -1;
168. }

170. /**
171. * Creates ordinal RDF resource out of an integer.
172. *
173. * @param    Integer    $num
174. * @return    object Resource
175. * @access    public
176. */
177. function createOrd($num)  {
178. return new Resource(RDF_NAMESPACE_URI . '_' . $num);
179. }

181. /**
182. * Prints a MemModel as HTML table.
183. * You can change the colors in the configuration file.
184. *
185. * @param    object MemModel     &$model
186. * @access    public
187. */
188. function writeHTMLTable(&$model)  {
189. $nms = $model->getParsedNamespaces();
190. $names = '';
191. $pre = '';


194. echo '<table border="1" cellpadding="3" cellspacing="0" width="100%">' . LINEFEED;
195. echo INDENTATION . '<tr bgcolor="' . HTML_TABLE_HEADER_COLOR . '">' . LINEFEED . INDENTATION . INDENTATION . '<td td width="68%" colspan="3">';
196. echo '<p><b>Base URI:</b> ' . $model->getBaseURI() . '</p></td>' . LINEFEED;
197. echo INDENTATION . INDENTATION . '<td width="32%"><p><b>Size:</b> ' . $model->size() . '</p></td>' . LINEFEED . INDENTATION . '</tr>';

199. echo '<tr><td><b>Prefix:</b>'.'<br/></td><td colspan="3"><b>Namespace:</b>'.'<br/></td></tr>';
200. $i=0;
201. if($nms != false){
202. foreach($nms as $namespace => $prefix){
203. if($i==0){
204. $col = HTML_TABLE_NS_ROW_COLOR0;
205. }else{
206. $col = HTML_TABLE_NS_ROW_COLOR1;
207. }
208. echo '<tr bgcolor="'.$col.'"><td>'.$prefix.'</td><td colspan="3">'.$namespace.'</td></tr>';
209. $i++;
210. $i%=2;
211. }
212. }else{
213. echo '<tr><td>-</td><td colspan="3">-</td></tr>';
214. }


219. echo INDENTATION . '<tr bgcolor="' . HTML_TABLE_HEADER_COLOR . '">' . LINEFEED . INDENTATION . INDENTATION . '<td width="4%"><p align=center><b>No.</b></p></td>' . LINEFEED . INDENTATION . INDENTATION . '<td width="32%"><p><b>Subject</b></p></td>' . LINEFEED . INDENTATION . INDENTATION . '<td width="32%"><p><b>Predicate</b></p></td>' . LINEFEED . INDENTATION . INDENTATION . '<td width="32%"><p><b>Object</b></p></td>' . LINEFEED . INDENTATION . '</tr>' . LINEFEED;

221. $i = 1;
222. foreach($model->triples as $key => $statement) {
223. $infered='';
224. if (is_a($statement,'InfStatement')) $infered='<small>(infered)</small>';
225. echo INDENTATION . '<tr valign="top">' . LINEFEED . INDENTATION . INDENTATION . '<td><p align=center>' . $i . '.<BR>'.$infered.'</p></td>' . LINEFEED;
226. // subject
227. echo INDENTATION . INDENTATION . '<td bgcolor="';
228. echo RDFUtil::chooseColor($statement->getSubject());
229. echo '">';
230. echo '<p>' .  RDFUtil::getNodeTypeName($statement->getSubject());
231. if(is_a($statement->subj,'Resource')){
232. $ns = $statement->subj->getNamespace();
233. if(isset($nms[$ns])){
234. echo $nms[$ns].':'.RDFUtil::getLocalName($statement->subj);
235. }else{
236. echo $statement->subj->getLabel();
237. }
238. }
239. echo '</p></td>' .  LINEFEED;
240. // predicate
241. echo INDENTATION . INDENTATION . '<td bgcolor="';
242. echo RDFUtil::chooseColor($statement->getPredicate());
243. echo '">';
244. echo '<p>' . RDFUtil::getNodeTypeName($statement->getPredicate());
245. if(is_a($statement->pred,'Resource')){
246. $ns = $statement->pred->getNamespace();
247. if(isset($nms[$ns])){
248. echo $nms[$ns].':'.RDFUtil::getLocalName($statement->pred);
249. }else{
250. echo $statement->pred->getLabel();
251. }
252. }
253. echo '</p></td>' .  LINEFEED;
254. // object
255. echo INDENTATION . INDENTATION . '<td bgcolor="';
256. echo RDFUtil::chooseColor($statement->getObject());
257. echo '">';
258. echo '<p>';
259. if (is_a($statement->getObject(), 'Literal')) {
260. if ($statement->obj->getLanguage() != null) {
261. $lang = ' <b>(xml:lang="' . $statement->obj->getLanguage() . '") </b> ';
262. } ELSE $lang = '';
263. if ($statement->obj->getDatatype() != null) {
264. $dtype = ' <b>(rdf:datatype="' . $statement->obj->getDatatype() . '") </b> ';
265. } ELSE $dtype = '';
266. } else {
267. $lang = '';
268. $dtype = '';
269. }
270. $label = $statement->obj->getLabel();
271. if(is_a($statement->obj,'Resource')){
272. $ns = $statement->obj->getNamespace();
273. if(isset($nms[$ns])){
274. $label = $nms[$ns].':'.RDFUtil::getLocalName($statement->obj);
275. }else{
276. $label = $statement->obj->getLabel();
277. }
278. }

280. echo  RDFUtil::getNodeTypeName($statement->getObject())
281. .nl2br(htmlspecialchars($label)) . $lang . $dtype;

283. echo '</p></td>' . LINEFEED;
284. echo INDENTATION . '</tr>' . LINEFEED;
285. $i++;
286. }
287. echo '</table>' . LINEFEED;
288. }

290. /**
291. * Chooses a node color.
292. * Used by RDFUtil::writeHTMLTable()
293. *
294. * @param    object Node    $node
295. * @return    object Resource
296. * @access    private
297. */
298. function chooseColor($node)  {
299. if (is_a($node, 'BlankNode'))
300. return HTML_TABLE_BNODE_COLOR;
301. elseif (is_a($node, 'Literal'))
302. return HTML_TABLE_LITERAL_COLOR;
303. else {
304. if (RDFUtil::getNamespace($node) == RDF_NAMESPACE_URI ||
305. RDFUtil::getNamespace($node) == RDF_SCHEMA_URI ||
306. RDFUtil::getNamespace($node) == OWL_URI
307. )

309. return HTML_TABLE_RDF_NS_COLOR;
310. }
311. return HTML_TABLE_RESOURCE_COLOR;

313. }

315. /**
316. * Get Node Type.
317. * Used by RDFUtil::writeHTMLTable()
318. *
319. * @param    object Node    $node
320. * @return    object Resource
321. * @access    private
322. */
323. function getNodeTypeName($node)  {
324. if (is_a($node, "BlankNode"))
325. return 'Blank Node: ';
326. elseif (is_a($node, 'Literal'))
327. return 'Literal: ';
328. else {
329. if (RDFUtil::getNamespace($node) == RDF_NAMESPACE_URI ||
330. RDFUtil::getNamespace($node) == RDF_SCHEMA_URI ||
331. RDFUtil::getNamespace($node) == OWL_URI)
332. return 'RDF Node: ';
333. }
334. return 'Resource: ';

336. }


339. /**
340. * Short Prefix for known and/or parsed Namespaces by given URI and Model
341. * Uses $default_prefixes defined in constants.php and getParsedNamespaces()
342. * Returns FALSE if no matching prefix is found
343. *
344. * @author   Anton K�stlbacher <anton1@koestlbacher.de>
345. * @param    string  $uri
346. * @param    object $model
347. * @return   string, boolean
348. * @access   public
349. * @throws   PhpError
350. */

352. function guessPrefix($uri, &$model)
353. {
354. global $default_prefixes;
355. $namespace = RDFUtil::guessNamespace($uri);
356. $par_nms   = $model->getParsedNamespaces();
357. if (isset($par_nms[$namespace]))
358. {
359. $prefix = $par_nms[$namespace];
360. }
361. else
362. {
363. $prefix = array_search($namespace, $default_prefixes);
364. }
365. if($prefix !== false)
366. {
367. return $prefix;
368. }
369. else
370. {
371. return false;
372. }
373. }


376. /**
377. * Generates a dot-file for drawing graphical output with the
378. * graphviz-application which can be downloaded at http://www.graphviz.org
379. * If the graphviz-application is installed and its path is set to the
380. * correct value in constants.php we can directly generate any
381. * file format graphviz supports, e.g. SVG, PNG
382. * Parameters: model to visualize, output format, use prefixes
383. *
384. * !!!!!!!!!!!!!!!!!!!!!!!!!!!!!!!!!!!!!!!!!!!!!!!!!!!!!!!!!!!!!!!!!!!!!!
385. * WARNING: Graphviz can be slow with large models.
386. * !!!!!!!!!!!!!!!!!!!!!!!!!!!!!!!!!!!!!!!!!!!!!!!!!!!!!!!!!!!!!!!!!!!!!!
387. *
388. * @author   Anton K�stlbacher <anton1@koestlbacher.de>
389. * @param    object  Model
390. * @param    string  $format
391. * @param    boolean $short_prefix
392. * @return   string, binary
393. * @access   public
394. * @throws   PhpError
395. */

397. function visualizeGraph(&$model, $format = "input_dot", $short_prefix = TRUE)
398. {
399. global $graphviz_param;
400. $i = 0;

402. foreach ($model->triples as $key => $statement)
403. {
404. $subject   = $statement->getLabelSubject();
405. $predicate = $statement->getLabelPredicate();
406. $object    = $statement->getLabelObject();

408. // format subject
409. if (!isset($attrib[$subject]))
410. {
411. if (is_a($statement->subject(),'BlankNode'))
412. {
413. $attrib[$subject] = $graphviz_param['BLANKNODE_STYLE'];
414. }
415. else
416. {
417. if ($short_prefix == TRUE && RDFUtil::guessPrefix($subject, $model) != FALSE)
418. {
419. $prefix = RDFUtil::guessPrefix($subject, $model);
420. $subject_label = $prefix.":".RDFUtil::guessName($subject);
421. $attrib[$subject] = "label=\"".$subject_label."\" ";
422. if(!isset($prefix_array[$prefix]))
423. {
424. $prefix_array[$prefix] = RDFUtil::guessNamespace($subject);
425. }
426. }
427. if (GRAPHVIZ_URI == TRUE)
428. {
429. $attrib[$subject] .= "URL=\"".$subject."\"";
430. }
431. }
432. }

434. // format predicate
435. if ($short_prefix == TRUE && RDFUtil::guessPrefix($predicate, $model) != FALSE)
436. {
437. $prefix = RDFUtil::guessPrefix($predicate, $model);
438. $predicate_label = "label=\"".$prefix.":".RDFUtil::guessName($predicate)."\"";
439. if(!isset($prefix_array[$prefix]))
440. {
441. $prefix_array[$prefix] = RDFUtil::guessNamespace($predicate);
442. }
443. }
444. else
445. {
446. $predicate_label = "label=\"".$predicate."\"";
447. }

449. if (is_a($statement,'InfStatement'))
450. {
451. $predicate_label .= " ".$graphviz_param['INFERRED_STYLE'];
452. }
453. else
454. {
455. if (GRAPHVIZ_URI == TRUE)
456. {
457. $predicate_label .= "URL=\"".$predicate."\"";
458. }
459. }

461. // format object
462. if (!isset($attrib[$object]))
463. {
464. if (is_a($statement->object(),'BlankNode'))
465. {
466. $attrib[$object] = $graphviz_param['BLANKNODE_STYLE'];
467. }
468. elseif (is_a($statement->object(),'Literal'))
469. {
470. $object_label = $object;
471. $object = "literal".$i;
472. $i++;
473. $attrib[$object] = "label=\"$object_label\" ".$graphviz_param['LITERAL_STYLE'];
474. }
475. else
476. {
477. if ($short_prefix == TRUE && RDFUtil::guessPrefix($object, $model) != FALSE)
478. {
479. $prefix = RDFUtil::guessPrefix($object, $model);
480. $object_label = $prefix.":".RDFUtil::guessName($object);
481. $attrib[$object] = "label=\"".$object_label."\" ";
482. if(!isset($prefix_array[$prefix]))
483. {
484. $prefix_array[$prefix] = RDFUtil::guessNamespace($object);
485. }
486. }
487. if (GRAPHVIZ_URI == TRUE)
488. {
489. $attrib[$object] .= "URL=\"".$object."\"";
490. }
491. }
492. }

494. // fill graph array
495. $graph[] = "\"".$subject."\" -> \"".$object."\" [".$predicate_label."];";
496. }

498. //generate DOT-file
499. $dot = "digraph G { ".$graphviz_param['GRAPH_STYLE']."\n edge [".$graphviz_param['PREDICATE_STYLE']."]\n node [".$graphviz_param['RESOURCE_STYLE']."]\n";
500. if (isset($attrib))
501. {
502. foreach ($attrib AS $key => $value)
503. {
504. $dot .= "\"$key\" [$value];\n";
505. }
506. }
507. if (!isset($graph))
508. {
509. $dot .= "error [shape=box,label=\"No Statements found!\"]";
510. }
511. else
512. {
513. $dot .= implode("\n", $graph);
514. }


517. if (GRAPHVIZ_STAT == TRUE)
518. {
519. $stat_label = "| ".$model->size()." Statements drawn";
520. }
521. if ((strstr($graphviz_param['GRAPH_STYLE'], 'rankdir="LR"') === FALSE) && (strstr($graphviz_param['GRAPH_STYLE'], 'rankdir=LR') === FALSE))
522. {
523. $sep1 = "}";
524. $sep2 = "";
525. }
526. else
527. {
528. $sep1 = "";
529. $sep2 = "}";
530. }

532. if ($short_prefix == TRUE && isset($prefix_array))
533. {
534. $struct_label = "{ { Base URI: ".$model->getBaseURI()." $sep1 | { { ".implode("|", array_keys($prefix_array))." } | { ".implode("|", $prefix_array)." } } $stat_label } $sep2";
535. }
536. else
537. {
538. $struct_label = "{ { Base URI: ".$model->getBaseURI()."$sep1 ".$stat_label." } }";
539. }

541. $dot .= "\n struct [shape=Mrecord,label=\"$struct_label\",".$graphviz_param['BOX_STYLE']."];\n";
542. $dot .= " vocabulary [style=invis];\n struct -> vocabulary [style=invis];\n}";

544. // if needed call dot.exe
545. if (($format != "input_dot") && (defined('GRAPHVIZ_PATH')) && (strstr(GRAPHVIZ_FORMAT, $format) !== FALSE))
546. {
547. mt_srand((double)microtime()*1000000);
548. $filename=GRAPHVIZ_TEMP.md5(uniqid(mt_rand())).".dot";
549. $file_handle = @fopen($filename, 'w');
550. if ($file_handle)
551. {
552. fwrite($file_handle, $dot);
553. fclose($file_handle);
554. }
555. $dotinput = " -T".$format." ".$filename;

557. ob_start();
558. passthru(GRAPHVIZ_PATH.$dotinput);
559. $output = ob_get_contents();
560. ob_end_clean();
561. unlink($filename);
562. echo $output;
563. return TRUE;
564. }
565. elseif ($format == "input_dot")
566. {
567. echo $dot;
568. return TRUE;
569. }
570. else
571. {
572. return FALSE;
573. }
574. }

576. } // end: RDfUtil

578. ?>
```

Documentation generated on Thu, 7 Jul 2005 13:43:08 +0200 by phpDocumentor 1.3.0RC3
